# Expanding connectomics to the laminar level: A perspective

**DOI:** 10.1162/netn_a_00304

**Published:** 2023-06-30

**Authors:** Ittai Shamir, Yaniv Assaf

**Affiliations:** Department of Neurobiology, Faculty of Life Sciences, Tel Aviv University, Tel Aviv, Israel; Sagol School of Neuroscience, Tel Aviv University, Tel Aviv, Israel

**Keywords:** Connectomics, Cortical layers, Computational models, Neuronal structures, Anatomical mapping, Brain network analysis

## Abstract

Despite great progress in uncovering the complex connectivity patterns of the human brain over the last two decades, the field of connectomics still experiences a bias in its viewpoint of the cerebral cortex. Due to a lack of information regarding exact end points of fiber tracts inside cortical gray matter, the cortex is commonly reduced to a single homogenous unit. Concurrently, substantial developments have been made over the past decade in the use of relaxometry and particularly inversion recovery imaging for exploring the laminar microstructure of cortical gray matter. In recent years, these developments have culminated in an automated framework for cortical laminar composition analysis and visualization, followed by studies of cortical dyslamination in epilepsy patients and age-related differences in laminar composition in healthy subjects. This perspective summarizes the developments and remaining challenges of multi-T1 weighted imaging of cortical laminar substructure, the current limitations in structural connectomics, and the recent progress in integrating these fields into a new model-based subfield termed ‘laminar connectomics’. In the coming years, we predict an increased use of similar generalizable, data-driven models in connectomics with the purpose of integrating multimodal MRI datasets and providing a more nuanced and detailed characterization of brain connectivity.

## INTRODUCTION

**Figure 1.**
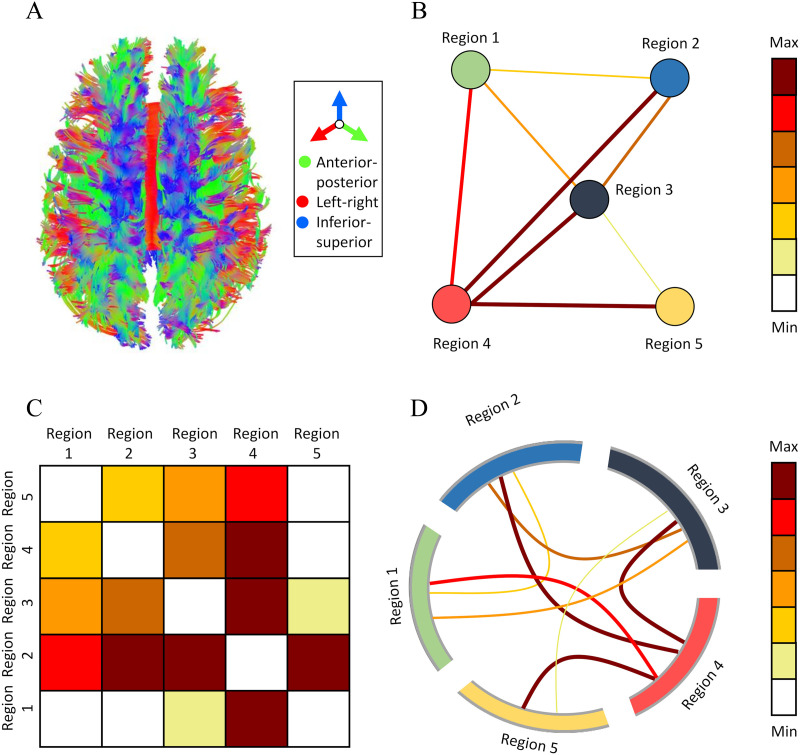
Various representations of structural connectomics. (A) Tractogram: a three-dimensional streamline diagram of fiber tract connections across the entire brain (color coded according to directions, seen from a top view). (B) Network graph: a graph representation consisting of nodes, signifying cortical regions (individually colored), and edges, signifying the strength of connections (colored according to heat map). (C) Connectivity matrix: a matrix representation, where value {i, j} signifies the strength of connections between cortical regions i and j (colored according to heat map). (D) Circular graph: a graph representation, where cortical regions are located across the circle’s circumference (individually colored), and the chords across its center signify the strength of connections (colored according to heat map).

Over the last two decades, the field of neuroimaging has made great strides in its exploration of the intricate patterns of interconnectivity of the human brain, led in part by the Human Connectome Project (Setsompop et al., 2013; Sporns et al., 2005; Van Essen et al., 2013). Structural connectomics using diffusion MRI (see Figure 1) has experienced significant developments in recent years (e.g., Assaf et al., 2019; Maier-Hein et al., 2017). These developments include investigation of the economy of connectivity of the human brain (Bassett & Bullmore, 2006, 2016; Bullmore & Sporns, 2012), exploration of its rich-club organization properties (van den Heuvel & Sporns, 2011), and identification of various central network hubs in the human brain (Sporns et al., 2007; van den Heuvel & Sporns, 2013). Recent studies have also explored intrahemispheric and interhemispheric wiring patterns in the human brain (Krupnik et al., 2021), and even patterns of wiring conservation across a variety of mammalian species (Assaf et al., 2020).

## CURRENT LIMITATIONS OF STRUCTURAL CONNECTOMICS

The field of structural connectomics still experiences conceptual and methodological drawbacks related to diffusion MRI techniques. Some of the main drawbacks on the conceptual side involve the fact that the dMRI measures displacement of water molecules, and not their diffusion directly. Furthermore, the measured displacement of water molecules does not always follow a Gaussian law, as previously assumed, since it is sometimes restricted in biological tissues.

If we focus on fiber tractography (FT), many methodological and technical pitfalls appear. Deterministic streamline tractography, the most widespread method for tractography, uses dMRI to compute the orientation density function (ODF) and then reduces it to vectors representing the strongest and most likely directional connections. The resulting model of the brain’s fiber tracts, also known as a tractogram, includes all the three-dimensional streamlines across the entire brain. Several studies have detailed the limitations relating to the inference of structural connectivity from local field potentials (Bastiani et al., 2012; Calamante, 2019; De Santis et al., 2014; Jeurissen et al., 2019; Jones et al., 2013), culminating in a recent international tractography challenge (Maier-Hein et al., 2017). The most prevalent limitations include the following:

- Strong effect of methodology: choice of methodology, including tractography algorithm, had a strong effect on resulting accuracy levels, revealing a tradeoff between sensitivity and specificity.
- High false positive rates: even the use of high-resolution images in FT still resulted in tractograms containing more invalid than valid (illogical and/or disconnected) fiber bundles.
- Errors in reconstructing strong tracts: many algorithms experienced high false positive rates when reconstructing strong tracts, due to increasingly high certainty assigned to strong local field potentials.
- Difficulty reconstructing small tracts: many FT algorithms also experienced high false negative rates for small tract with diameters of 2 mm or less, such as the anterior commissure (CA).
- Difficulty reconstructing complex geometry: the intricate geometry of many fiber junctions posed a source of many artifacts and miscalculations in FT (crossing fibers within a single voxel).

Lastly, one of the biggest methodological limitations in tractography and structural connectomics in general is the difficulty in estimating the exact terminations of fiber tracts inside cortical gray matter. The cortical termination bias involves difficulties in estimating tract terminations not only laterally across the cortical folding (gyral bias), but also radially across the cortical layers. The main causes for this significant limitation are the partial volume effect associated with voxel-wise FT approaches, coupled with the difficulties in estimating small and complex fiber tracts.

Over the years many advanced techniques for tractography have been proposed for tackling these limitations in structural connectomics. Some of these advancements include techniques for resolving high false positive connections using filtering, replacing manual filtering based on prior knowledge with thresholding of connectivity matrices or with automated knowledge and data-driven algorithms (Zhang et al., 2022). Other advancements have focused on techniques for either handling strong tracts by increasing specificity, or for handling small tracts by increasing sensitivity (Zhang et al., 2022). The gyral bias in estimating fiber tract terminations is commonly addressed using targeted tracking, which defines regions for inclusion and exclusion based on prior anatomical constraints (Yeh et al., 2021).

It should be noted that despite the many technical developments in the field, there still lacks a single ‘gold standard’ tractography methodology. Furthermore, the estimation of exact terminations of fiber tracts in cortical gray matter remains a significant difficulty that has resulted in a biased representation of the cerebral cortex. This representation falsely considers the cortex a single homogenous unit and ignores its microstructural laminar composition.

## HISTOLOGICAL EXPLORATIONS OF LAMINAR CONNECTIVITY

**Figure 2.**
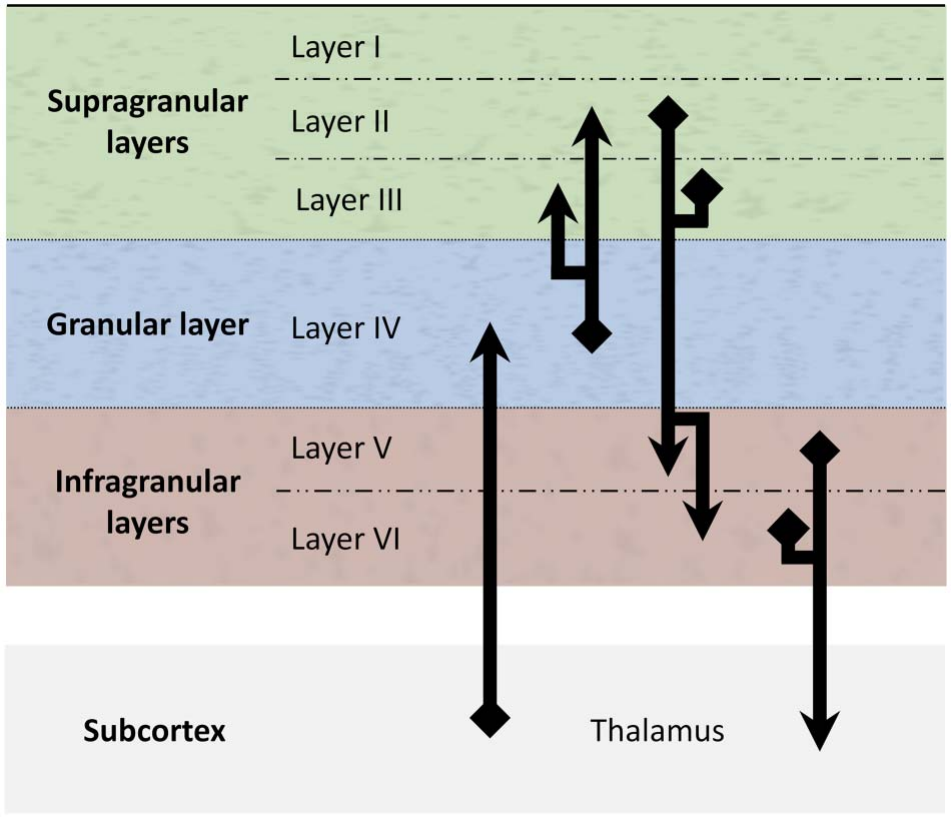
A schematic representation of the canonical microcircuit: The three laminar components (supragranular, granular, and infragranular), consisting of six cortical layers (I–VI), are interconnected, and connected to and from the thalamus (in the subcortex) according to this detailed connection scheme. It is worth noting that this is a simplified representation of the canonical microcircuit. This version of the microcircuit omits cortico-cortical connections of all kinds, including feedforward, feedback, and lateral connections. It is also a partial representation of intracolumnar connections that excludes some connections that are often added, such as a recurrent connection from layers V/VI to layer IV.

Following the partitioning of the cortex into cortical layers at the beginning of the 20th century, the cortical column was introduced as a single unit that spans across all six layers and repeats across the brain (Hubel & Wiesel, 1959, 1962; Lorente de Nó, 1949; Mountcastle, 1957). The concept of the cortical column stemmed from Hubel and Wiesel’s exploration of the mammalian visual cortex, which resulted in the discovery of ocular dominance columns (Hubel & Wiesel, 1969). After the cortical column, the canonical microcircuit was introduced as a connectivity unit across and between the cortical layers (Gilbert & Wiesel, 1983; Hellweg et al., 1977; Szentágothai, 1975). The term ‘canonical microcircuit’ was coined by Douglas et al. (1989) and it entails the following basic scheme (Dhruv, 2015): thalamic input to granular layer IV, layer IV projections to layers II and III, projections from layers II and III to layers V and VI, and finally projections from layers V and VI back to the thalamus (see Figure 2).

Since then, a variety of methods have been applied outside the field of neuroimaging in the exploration of connectivity patterns of the brain on the cortical layer level, including histological staining, tract tracing (retrograde and anterograde), electrical recordings, and others. Most of these explorations used animal models, ranging from cats (Douglas et al., 1989; Hubel & Wiesel, 1962) to nonhuman primates such as the rhesus macaque (Felleman & Van Essen, 1991; Mountcastle, 1997; Rockland & Pandya, 1979). Most of the studies of layer connectivity have focused on specific cortical regions, primarily the visual cortex or other regions such as the somatosensory, motor, or auditory cortices (Shamir & Assaf, 2021).

This approach to cortical layer connectivity has since been reexamined and the inherently interlinked concepts of the cortical column and the cortical microcircuit have been questioned (Feldmeyer, 2012). What followed were studies that explored layer connections using a broader approach, with one such repeatedly reported model offering rules of interregional cortical layer connections based on the granularity indices of the connecting regions (Barbas, 1986; Barbas & Rempel-Clower, 1997; Beul & Hilgetag, 2015; Shipp, 2005; von Economo, 2009). Broader, integrative studies have also been performed on hundreds of published studies on layer connectivity in specific regions of the primate cortex (Schmidt et al., 2018; Solari & Stoner, 2011), and even comparing layer connectivity between different regions and different species (DeFelipe et al., 2002).

## RECENT DEVELOPMENTS IN IMAGING THE CORTICAL LAYERS

Since its discovery at the beginning of the 20th century (Garey, 2006), the geometrically complex and highly organized laminar structure of the cerebral cortex has been assumed to play an integral role in the development and function of the human brain, as well as different pathologies of the brain (Kiernan & Rajakumar, 2014). Until relatively recently, the intricate laminar composition of the cerebral cortex has been assumed to be beyond the resolution capabilities of MRI neuroimaging.

Prior to this progress, it was already established that myelination causes shortening of T1 values. In 1992, a study of both in vivo and postmortem human brains used high-resolution T1-weighted images to identify the striate cortex and revealed six laminar clusters within it, with decreasing T1 values from the outermost to the innermost parts of the cortex (Clark et al., 1992). Consequently, T1-weighted MRI techniques have successfully segmented cortical gray matter from bordering white matter and cerebrospinal fluid (Fischl, 2012; Kiernan & Rajakumar, 2014; Scholtens et al., 2015), further solidifying the link between T1 values and myeloarchitecture (Van Essen et al., 2019).

Since then, studies have offered a variety of MRI approaches for exploring cortical gray matter on the laminar level, including T1-, T2-, and T2*-weighted images, as well as R1, R2, and R2* susceptibility images. Barbier et al. (2002) used high-resolution T1-weighted imaging at 3T to delineate the striate cortex based on myelin content. Bridge and Clare (2006) showed a correspondence between delineations of the primary visual cortex by both high-resolution MRI and fMRI. Duyn et al. (2007) used high field at 7T to explore cortical substructure based on signal phase. Deistung et al. (2013) compared ultraresolution quantitative susceptibility maps with conventional gradient echo imaging techniques, including magnitude, phase, and R2* imaging. Lutti et al. (2014) used high-resolution quantitative mapping of R1 as a measure of cortical myelination. Glasser et al. (2014) reviewed a variety of methods for examining cortical myeline content, including T1 imaging, T2 imaging and even positron emission tomography (PET). Shafee et al. (2015) used the ratio of T1-weighted to T2-wieghted images to explore gray matter lamination. A recent study even explored diffusion MRI as a potential method for imaging the laminar substructure of cortical gray matter (Assaf, 2019). Of the modalities examined, T1, T2, and T2* imaging initially gained the most attention. Since then, the biggest challenge has been finding the most robust and precise modality that does not involve noisy acquisition, as is the case in T2* imaging, or long scan times and a pronounced partial volume effect, as is the case in T2 imaging.

The T1-weighted approach has proved most suitable so far, thanks to findings from a series of follow-up studies over the past decade. In 2012, a study used T1-weighted imaging to characterize the cortical layers in vivo across the entire brains of both human subjects and rat models (Barazany & Assaf, 2012). The study reestablished the correspondence between T1 clusters and the cortical layers by comparing the T1 clusters to histological findings in the rat brains. This study was followed by a larger scale study of both rat and human brains, using the same inversion recovery (IR) MRI protocol and a higher resolution version of the same protocol (Lifshits et al., 2018). The study demonstrated that the sound-to-noise ratio and partial volume effect involved in imaging layers with dimensions down to tens of microns make it unlikely to resolve the layers using high-resolution imaging. It concluded that the cortical layers are better separated using low-resolution multi-T1 mapping (high resolution in the relaxation domain), compared to high resolution in the image domain. In 2019, a comprehensive framework was presented for cortical laminar composition analysis using low-resolution multi-T1 mapping (Shamir et al., 2019). The study offered a whole-brain automatic methodology for analyzing and visualizing the laminar substructure of the cortex. The study tackled the partial volume effect associated with lower resolution images and implemented a spherical sampling system as a rotationally invariant alternative to cortical normals, which addressed the issue of normal miscalculations due to small inaccuracies in estimating cortical surfaces.

In 2021, a study used the same low-resolution multi-T1 weighted protocol to explore patterns of cortical dyslamination in epilepsy patients (Lotan et al., 2021). The study focused on epileptic patients with both focal cortical dysplasia (FCD) and periventricular nodular heterotopia (PNH), successfully revealing T1-layer specific cortical laminar abnormalities associated with the pathology. In 2022, a follow-up study used the same methodology to explore age-related differences across a group of 200 healthy subjects (Tomer et al., 2022). The study reestablished the validity of the framework for cortical laminar composition analysis and demonstrated its ability to capture differences in compositions across subject groups and brain regions.

It should be noted that despite the significant progress and success of the low-resolution multi-T1 approach for laminar composition analysis, it does have certain limitations. The first and most notable limitation is that T1 relaxation is still not considered a direct measure of cytoarchitecture, and as a result the approach is considered an indirect way of imaging and measuring the cortical layers. However, since T1 is considered a measure of myeloarchitecture (myeline content) and its correspondence with the cortical layers has been established (Barazany & Assaf, 2012; Lifshits et al., 2018; Lotan et al., 2021; Shamir et al., 2019; Tomer et al., 2022), the term ‘T1 layers’ is used instead in this context. An additional limitation relates to high-resolution variations in laminar composition within smaller examined cortical regions and within the cortical folding, which are restricted in this approach due to its low-resolution nature. Lastly, because this methodology is relatively novel and new, it has yet to be implemented and explored in large-scale populations studies (upwards of 1,000 subjects).

Nevertheless, it is our assertion that the T1 layer methodology can be solidified as a framework for direct measurement of cortical cytoarchitecture if a specific algorithmic challenge is overcome. This challenge involves spatial clustering of the resulting multidimensional, surface based T1 layer composition on a whole-brain scale and across a group of subjects. If successful, the T1 clusters will show both visual and quantitative correspondence with established cytoarchitectonic atlases and highlight the role of T1 imaging as a direct probe of tissue microstructure.

## INTEGRATING CORTICAL COMPOSITION INTO STRUCTURAL CONNECTOMICS

As the field of connectomics (and structural connectomics in particular) advanced, the need grew for a unified view of connectivity from the macroscale to the microscale (Rockland, 2019). Several studies have pointed to the potential of integrating microstructural information regarding cortical laminar composition with macrostructural information regarding cortical connectivity (Jbabdi & Johansen-Berg, 2011; Johansen-Berg, 2013). Subsequently, the extensive progress of the past two decades in the field of structural connectomics, coupled with the developments of recent years in imaging the cortical laminar substructure, have opened the door to new avenues in connectomics.

**Figure 3.**
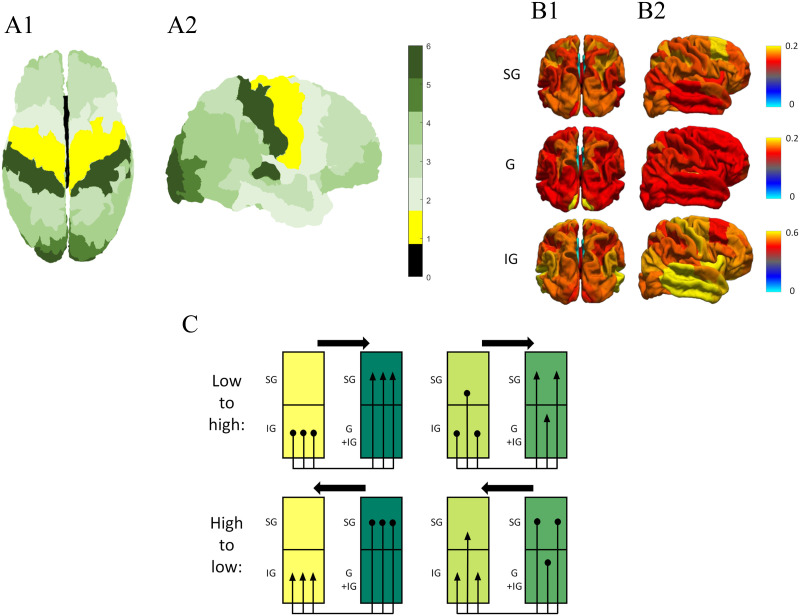
Components of the model of cortical laminar connectivity. The MRI-based, data-driven model formed by Shamir and Assaf (2021) offers a way to expand the structural connectome to the laminar level using the following components. (A) Granularity atlas: a cytoarchitectonic atlas that labels cortical regions according to the level of cellular granularity observed histologically, as reported by von Economo (2009) and further discussed by Beul and Hilgetag (2015) and by Scholtens et al. (2016). The indices: 0, allocortex; 1, agranular; 2, slightly granular; 3, 4, 5, increasingly granular; 6, granular. The atlas is seen from a top view (A1) and from a lateral right view (A2). (B) Laminar composition: a summary of laminar components across the cortex, based on data from Scholtens et al. (2016). The components: SG, supragranular component (layers I, II, and III); G, granular component (layer IV); IG, infragranular component (layers V and VI). The compositions are seen from a top view (B1) and from a lateral right view (B2). (C) Model rules of laminar connectivity: a schematic representation of the rules of horizontal connectivity, based on the granularity indices of the connecting regions as well as their laminar composition (Shamir & Assaf, 2021). Additional rules, regarding radial connectivity, are derived from canonical microcircuit (as seen in Figure 2).

In 2021, a study addressed this subject by presenting an MRI-based, data-derived model of cortical laminar connectivity that offers a way to overcome the biased representation of the cerebral cortex as a single homogenous unit (Shamir & Assaf, 2021). The study addressed the lack of information about exact terminations of fiber tracts inside the cortex by systematically reviewing 51 previously published histological studies that focused on patterns of brain connectivity on the cortical laminar level. A simplified model was then formed (Shamir & Assaf, 2021), consisting of a set of data-derived rules for expanding standard connectomics to the laminar level using the laminar composition of the cortex. The model consists of two principal types of connections: horizontal and radial connections. Horizontal connections include interregional connections within and between the hemispheres. Radial connections include intraregional connections within the cortical microcircuit as well as connections to the subcortex. All connections are expansions of findings from standard connectomics, except for connections within the microcircuit, which are assumed connections. Model rules are applied according to the granularity indices of the connecting regions and weighted according to their laminar compositions (see Figure 3).

A follow-up validation study was then performed, implementing the same model on multimodal MRI datasets of the macaque brain (Shamir & Assaf, 2022). The macaque was chosen specifically with the intent to compare the resulting model of cortical laminar connectivity to the connectivity patterns established through a tract tracing study of the macaque visual cortex (Felleman & Van Essen, 1991). The study further validated the model of cortical laminar connectivity by reporting an accuracy level 83%, surprisingly high considering the methodological disparities between the two studies (and the 30-year gap).

**Figure 4.**
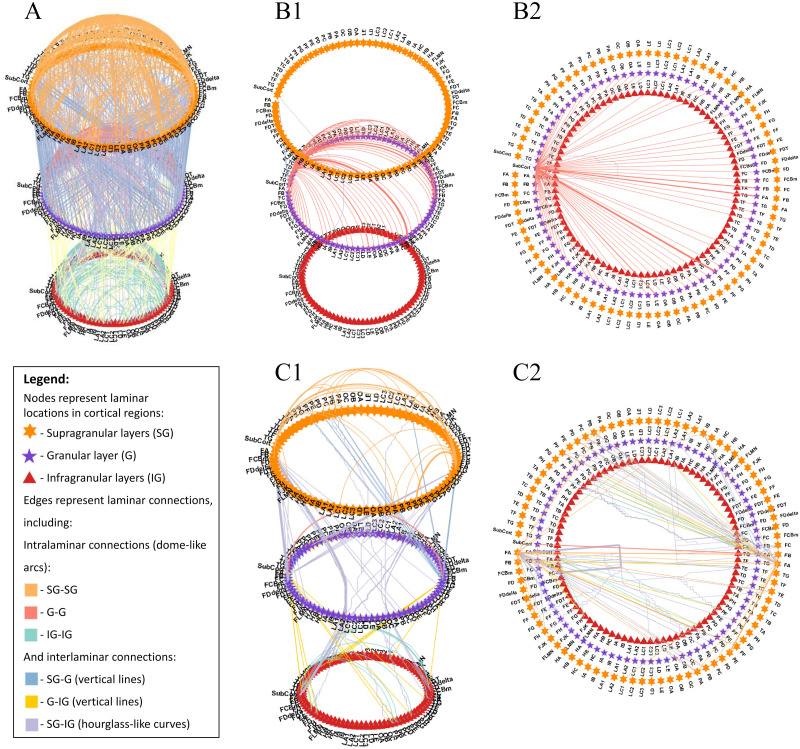
A multilayered, multidimensional circular network graph representing the laminar connectome of a healthy human subject across von Economo-Koskinas atlas regions. (A) All connections of the cortical laminar connectome, exhibiting the highly interconnected and complex nature of this model of laminar connections. (B) Laminar connections that include the subcortex from a side view (B1) and from a top view (B2), presenting the dominance of the granular layer in connections that include the subcortex (SubCort) and its high connectivity across all regions. (C) Laminar connections that include the primary motor cortex (M1) from a side view (C1) and from a top view (C2), presenting intricate patterns of laminar connections that include the precentral gyrus (FA) that roughly follow the canonical microcircuit: thalamic input to the granular layer, followed by projections to supragranular layers, which project deeper to infragranular layers, and then back to the thalamus. Connections are colored according to connection type, based on the connecting laminar groups, where: supragranular layers include T1 layers 1–3 (top circle-orange pentagons), granular layer includes layer 4 (mid circle-purple stars), and infragranular layers include layers 5 and 6 (bottom circle-red triangles). Data taken from Shamir and Assaf (2021), freely available at https://github.com/ittais/Circular-Connectome, using the modeling functions from Shamir and Assaf (2022), freely available at github.com/ittais/Laminar_Connectivity.

In 2022, following the formation and corroboration of this model of laminar connectivity, a study modeled the laminar connectome of 30 healthy human subjects (Shamir et al., 2022). Unlike the case of the macaque visual cortex, no single gold standard of laminar connectivity exists for the human brain. Nonetheless, the study examined the resulting networks of laminar connectivity and found several expected points of reference, including high centrality of granular connections (involving T1 layer IV) to visual, motor, and auditory regions (see Figure 4).

It should be noted that the model of cortical laminar connectivity presented in these studies (Shamir & Assaf, 2021, 2022; Shamir et al., 2022) has certain limitations. The first major limitation is that the model is a simplified model, which reduces cortical components, including the reduction of six T1 layers to three laminar components, with the purpose of increasing the dimensionality of the connectome. The second major limitation is that the model does not estimate the probability of these laminar-level connections, since they are applied via a set of predefined rules of connectivity.

Taking these limitations into account, alongside the sanity checks found in the visual, motor, and auditory cortices, the model offers a more nuanced whole-brain look at patterns of connectivity on the laminar level. This characterization of the healthy human laminar connectome could provide a new level of detail in the examination of different groups of interest that are presumed to involve distinct features of layer-dependent connectivity.

## SUMMARY

Over the past 20 years, the field of neuroimaging has experienced great advancements in imaging both white matter connectomics and more recently also in imaging the laminar composition of gray matter. The progress in imaging gray matter has amplified the demand for a less biased representation of the cerebral cortex in connectomics that addresses the lack of information regarding the exact terminations of fiber tracks inside the cortical foldings.

To address this issue and overcome this bias, knowledge-based models must be used. The MRI-based, data-driven model discussed here (Shamir & Assaf, 2021, 2022; Shamir et al., 2022) offers one such model by using previously published histological findings to form a simplified model of cortical laminar connectivity. Models of this kind open the door to a new subfield of structural connectomics termed laminar connectomics. Currently, the field of connectomics includes three intertwining subfields, each focused on a different type of brain connectivity: structural, functional, and effective (Park & Friston, 2013). Effective connectomics uses the anatomical constraints of structural connectivity in dynamic causal models to explain the functional signal and explore patterns of activation between different cortical regions (Friston et al., 2003, 2019). In other words, the novel subfield of laminar connectomics is a data-driven enhancement of the resolution of anatomical connections, in the same sense that effective connectomics is an expansion of functional connectomics.

While the model discussed here has its own set of drawbacks and limitations, it offers a straightforward approach to integrating multimodal MRI datasets into a whole-brain view of connectivity on the laminar level. Moreover, this model showcases the benefits of using data-driven models to explore structural connectomics in a more unbiased and nuanced approach, which considers the laminar structure of the cortex. We believe that the use of such novel models will play an important role in structural connectomics even if higher resolution imaging of connectivity is achieved, on the condition that the models are knowledge-based, applicable, and generalizable to any existing or future structural connectivity methodology. The resulting modeled multilayered connectomes will necessitate the use and development of advanced network analysis tools that can visualize and explore multidimensional networks (such as muxViz, De Domenico et al., 2015).

The potential of this expansion of structural connectomics is wide: now that a basic characterization of the healthy human laminar connectome has been achieved, this detailed model of laminar-level connections can be used to explore different pathologies. This framework, or others like it, can be implemented to explore pathologies that are assumed to entail abnormalities in laminar-level structure and connections, such as autism spectrum disorder (ASD) or schizophrenia. This framework can also be used to enhance the exploration of the mechanisms behind different motor, auditory, or visual skills. The use of multidimensional complex network analysis tools could shed new light on the connectivity patterns and mechanisms that make these groups distinct on the laminar level.

## AUTHOR CONTRIBUTIONS

Ittai Shamir: Conceptualization; Data curation; Formal analysis; Investigation; Methodology; Project administration; Software; Supervision; Validation; Visualization; Writing – original draft; Writing – review & editing. Yaniv Assaf: Conceptualization; Data curation; Funding acquisition; Investigation; Methodology; Project administration; Resources; Supervision; Validation; Writing – review & editing.

## TECHNICAL TERMS

Human Connectome Project: A project sponsored by the National Institutes of Health (NIH), intended to map the connections of the healthy human brain.
Connectomics: A subfield of neuroscience focused on exploring the map of connections in the nervous system.
Diffusion MRI: A magnetic resonance imaging (MRI) method for measuring the displacement, or diffusion, of water molecules in various tissues.
Rich-club organization: A network phenomenon that involves the tendency of high-degree nodes to also be highly interconnected.
Network hubs: Nodes that occupy central positions in the general organization of a network.
Intrahemispheric: Connections within a single hemisphere (left or right) of the brain.
Interhemispheric: Connections between the two hemispheres (left and right) of the brain.
Orientation density function (ODF): A function used for describing the directionality of complex fiber architecture in the nervous system based on diffusivity.
Crossing fibers: A common problem in tractography involving multiple fiber bundles with significantly varying orientations.
Myeloarchitecture: The microscopic study of the arrangement of nerve fibers based on their myeline content.
T1-weighted imaging: A magnetic resonance imaging (MRI) pulse sequence that measures the longitudinal relaxation times, or T1 values, of various tissues.
T2-weighted imaging: A magnetic resonance imaging (MRI) pulse sequence that measures the transverse relaxation times, or T2 values, of various tissues.
T2*-weighted imaging: A magnetic resonance imaging (MRI) pulse sequence that measures the effective transverse relaxation times, or T2* values, of various tissues.
Cytoarchitecture: The microscopic study of the cellular structure, or architecture, of various tissues in the nervous system.
Multimodal MRI: A combination of different magnetic resonance imaging (MRI) sequences, or modalities.
